# Characterization of the electrical conductivity of bone and its correlation to osseous structure

**DOI:** 10.1038/s41598-018-26836-0

**Published:** 2018-06-05

**Authors:** Thomas Wyss Balmer, Soma Vesztergom, Peter Broekmann, Andreas Stahel, Philippe Büchler

**Affiliations:** 1 Institute for Surgical Technology and Biomechanics, University of Bern, Stauffacherstrasse 78, 3014 Bern, Switzerland; 2 Department of Physical Chemistry, Eötvös Loránd University, Pázmány Péter Sétány 1/A, 1117 Budapest, Hungary; 3 Department of Chemistry and Biochemistry, University of Bern, Freiestrasse 3, 3012 Bern, Switzerland; 4 School of Engineering, Bern University of Applied Sciences, Quellgasse 21, 2501 Biel, Switzerland

## Abstract

The interaction of osseous tissue with electric fields is an important subject. The electrical stimulation of bone promotes osteogenesis, while bone impedance has been proposed as a measure of osteoporosis, to follow fracture healing, or as a method to improve safety of surgical procedures. However, a deeper understanding of the electrical properties of bone and their relation to the architecture of osseous tissue is required to extend the range of use of electrical measurements to clinical studies. In this paper we apply electrical impedance spectroscopy to study the conductivity of fresh bovine tibia and we correlate the measured conductivities with its structural properties. Impedance was measured using a custom-made cell and a potentiostat. Bone conductivity was determined at 100 kHz, where the phase shift was negligible. A good agreement (R^2^ = 0.83) was found between the measured conductivity and the bone volume fraction, determined on microCT images. Based on this relationship, an equivalent circuit model was created for bone samples. The results of this *ex-vivo* study are comparable to previous *in-vivo* observations reporting bone resistivity as a function of bone density. This information can be used to construct a map of the tissue resistivity directly derived from clinical images.

## Introduction

The electrical stimulation of bone has been applied for more than a decade to stimulate bone growth in the case of nonunion^1,2^ or after spinal fusion^3,4^. Pulsed electromagnetic fields have been used to prevent bone loss in immobilized patients^5^. Electrical measurements have also been proposed as a tool that can detect pathological changes in the composition of the bone and might, therefore, be used as a diagnostic tool^6^. Electrical measurements, for example, might be used to detect loss of trabecular structure associated with osteoporosis, which cannot be seen in radiographic images. Furthermore, the electrical properties of bone play an important role in neuro-monitoring. This technique is used in surgery to warn the surgeon when their surgical instruments are in close proximity to critical nerves, allowing them to avoid damaging these important structures. The technique relies on the electrical stimulation of the nerve by using an electrode placed on the surgical tool^7–10^. As the instrument approaches the nerve, the electrical current induced by the electrode triggers a measurable response that depends largely on the inhomogeneous structure of the bone. Clinically, electromyography is used to measure the response of the muscle innervated by the target nerve, while the patient is under general anesthesia. For these reasons, it is important to understand the electrical properties of the bone and their relation to the underlying bone density and structure. This basic experimental work is required to develop and improve medical devices relying on the bone response to electric stimuli.

The accurate quantification of the electrical properties of the bone is challenging. Osseous tissue can be considered as an inhomogeneous and highly anisotropic material that contains less conductive bone mineral (a form of hydroxyapatite) and more conductive soft tissue such as marrow, also containing blood vessels and other bodily fluids. In order to determine its conductivity, bone tissue must be brought into contact with electronic conductors. This contact unavoidably results in the appearance of a capacitive term in the measured impedance spectra. Thus, it complicates the interpretation of measurements as well as the establishment of a good correlation between the electrical properties and the structure of osseous tissue.

Over the past four decades, several investigations have been performed in order to determine the electrical properties of osseous tissue – mostly conductivity and permittivity – across a broad range of frequencies^11–17^. These studies all suffer from limitations that are due to the (mostly capacitive) response of the metal/bone interface. Furthermore, these studies do not investigate the relationship between electrical properties and bone morphology.

Recently, Sierpowska *et al*.^6,18–21^. investigated the link between the electrical properties of bone and its mechanical characteristics^19^ and microstructure^20^. Relationships were established for the electrical permittivity and loss factor but not for the electrical conductivity, which the authors then associated with the density and water content of trabecular bone^6^. Their interpretation, however, relies on the assignment of measured phase shifts to the bulk properties of the studied conductive medium (osseous tissue), and surface effects are not taken into account. In this context, it is very important to note that the presence of phase boundaries (*i.e*., electrode/bone boundaries) does introduce a capacitive behavior to the measured impedance and that this interfacial contribution should not be confused with the bulk properties of osseous tissue. The effect of the interfacial capacitance cannot be cancelled by the application of a conductive gel, although this can indeed assure “better contact” between the bone sample and the electrode head. Consequently, in our measurements we attempted to minimize the phase shift that can arise at lower frequencies (<100 kHz) from the above-mentioned capacitive behavior of the electrode/bone boundary, and at higher frequencies (>100 kHz) from the stray capacitance of the measurement system.

The objective of this study was to quantify the relationship between bone conductivity and bone microstructure. Our hypothesis is that bone has a dominantly resistive behaviour and that any phase shift in the measurements can be attributed to interface effects^22^ or to stray capacitance. Therefore, special electrodes were designed to reduce stray capacitance and the conductivity of bone was determined at frequencies corresponding to a minimal phase shift.

## Materials and Methods

### Sample Preparation

**Figure 1:**
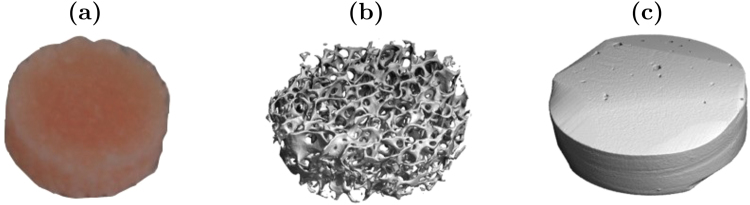
Cylindrical samples were cut out of bovine tibia (**a**). After measurement, each sample was imaged using a microCT scanner to quantify bone morphology. Both trabecular (**b**) and cortical (**c**) samples were included in this study representing a broad range of the bone volume to total volume ratio, *BV/TV*.

Twenty cylindrical bone samples were extracted from the proximal part of adult bovine tibia (Fig. 1). The samples were prepared by sectioning bone pieces of 20 mm length below the intercondylar eminence. A custom-made handsaw made of two parallel blades was used to obtain samples having two parallel faces. A hollow drill was then used to extract cylinders of 8 mm diameter from these sections. Drilling was performed within a water bath to avoid heating of the sample and any loss of water content. The process resulted in cylindrical samples of 20 mm in length, with axes aligned with the main orientation of the tibia. Therefore, most of the marrow canals proceeded axially, connecting both cylinder bases. The cylinders were further divided into shorter samples of lengths between 0.4 mm and 1.5 mm. The sectioning was performed using a diamond bandsaw (EXAKT GmbH, Norderstedt Germany). The bone samples were mounted on a precision vise to position them before cutting. Bone cutting was done at a very low feed rate to guarantee that the fatty marrow tissue remains inside the bone samples. Prior to conductivity measurements, the samples were stored in Ringer’s solution to avoid water loss. This solution was identical to the physiological solution used to clean operative fields during surgery. The whole measurement workflow – bone collection, preparation and measurement – was completed within 48 hours following the sacrifice of the animal.

### Electrical Impedance Measurements

**Figure 2:**
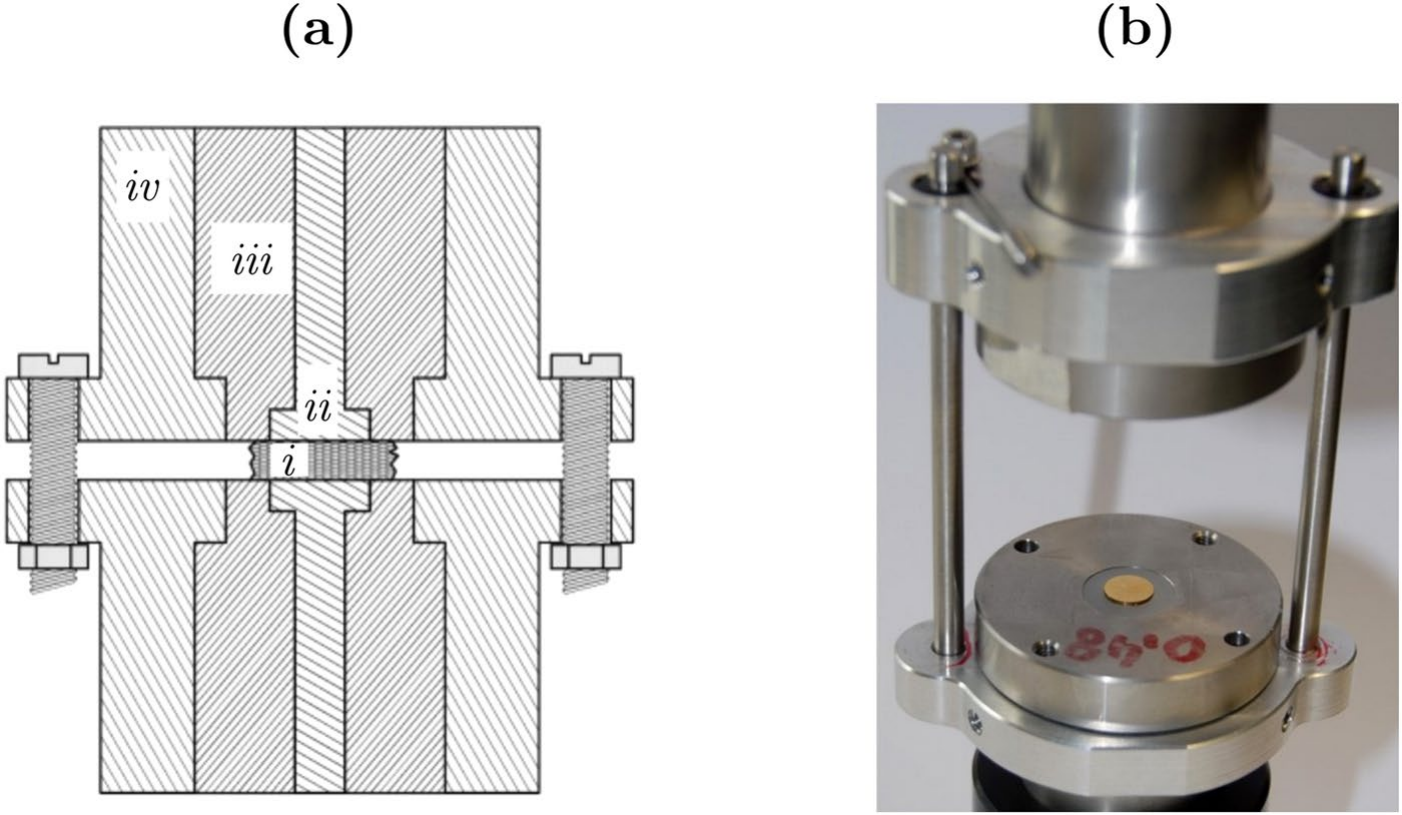
Sketch (**a**) and photograph (**b**) of the cell used for the measurement of bone impedance. The experimental setup is designed to reduce stray capacitance. The bone sample (i) is placed between the two gold electrodes (ii). An insulating Teflon separator (iii) is placed between the measurement electrodes and the stainless steel external shielding (iv). The external shielding of the upper and lower electrodes is connected and grounded.

Electrical properties were measured using a custom-made cell specifically designed to limit stray capacitance (Fig. 2). The cell was made of two stainless steel electrode holders to provide shielding of the electromagnetic noise. The upper and the lower metallic holders were connected together and to the ground. The upper holder was guided by roller bearings to freely move vertically along the rods connecting the upper and lower electrode holders. This guarantees a constant contact pressure on the samples corresponding to the weight of the holder (pressure of 96 kPa). The electrode heads were placed in the middle of the holders, isolated from the outside shielding by a Teflon cylinder. The very low permittivity of Teflon (*ε*_r_ ≈ 3) provides an additional protection against parasitic capacitance. The surfaces of the electrode heads contacting the bone sample were gold-coated in order to limit their corrosion.

Impedance spectra were recorded using an Autolab PGSTAT 302N potentiostat (Metrohm, Switzerland). Sinusoid voltage excitation signals of a 10 mV amplitude were applied at a DC setpoint of 0 V in a frequency range from 10 Hz to 1 MHz, at 50 logarithmically distributed frequencies. The total measurement time was about 150 seconds per sample.

To avoid any water loss of the samples, a fully water-saturated environment was created. A PMMA box was placed around the electrode system and a “cold humidifier” was used to maintain a level of humidity of 90% around the samples. During the experiment, the level of humidity and the temperature were constantly monitored and recorded using a temperature and humidity data-logger (ELV USB UTDL10, ELV Elektronik AG, Germany).

### Morphometric Characterization

Following electrical characterization, all the bone samples were scanned with a microCT scanner at a resolution of 16 µm (Scanco 40, Scanco, Switzerland). These high-resolution images were segmented using a threshold of 220 mgHA/cm^3^ to evaluate the following morphometric parameters: bone volume fraction (*BV/TV*), bone surface fraction (*BS/BV*), trabecular thickness (*TbTh*), trabecular spacing (*TbSp*), connectivity (*ConnD*), structure model index (*SMI*), trabecular number (*TbN*), mean density of the total volume (*MDTv*), and the geometrical degree of anisotropy (*DA*). These parameters were directly obtained from the analysis software provided by the manufacturer of the microCT (Image Processing Language IPL, Scanco, Switzerland). MicroCT images were also used to precisely determine the dimension of the cylindrical bone samples and to verify that the bone marrow remained inside the trabecular samples.

## Results

**Figure 3:**
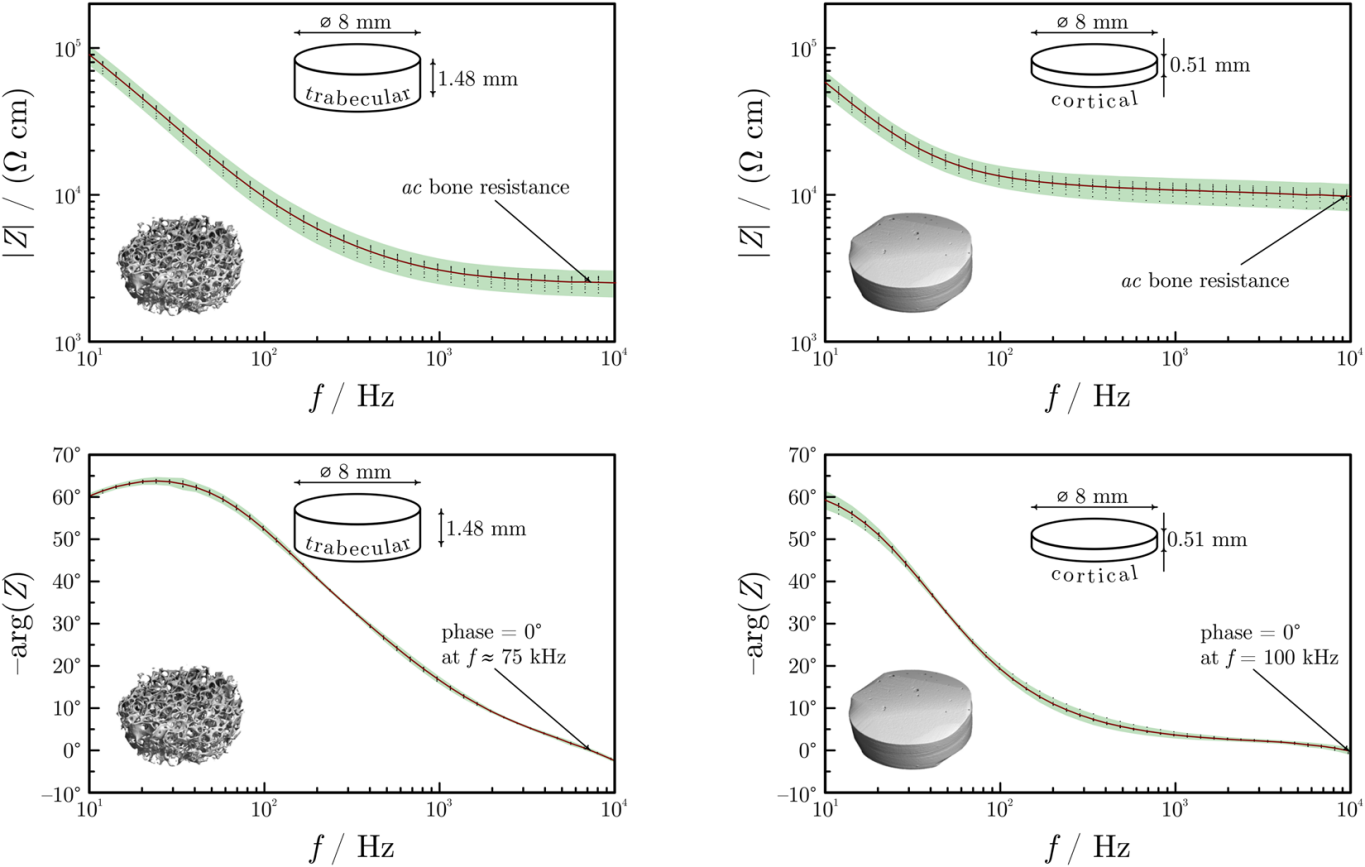
Representative impedance spectra shown on Bode plots (magnitude vs. frequency and phase shift vs. frequency plots) for a trabecular (left-hand side) and a cortical (right-hand side) sample. The inter-measurement deviation is small, showing a good reproducibility of the spectra. Impedances reach a more or less constant value at about 10 kHz. Readings were made at zero phase for all samples.

The impedance of 20 bone samples was measured. As expected, measurements showed a strong frequency dependence; the impedance was about 100 times higher at low frequencies than at high frequencies. This decrease is associated with a significant change of the phase shift. The phase is about 60° at low frequencies and drops to values close to zero when the frequency increases. Representative impedance *vs*. frequency plots obtained by multi-sample averaging for cortical and trabecular bone are shown in Fig. 3.

For the subsequent analysis, the bone impedance was determined at frequencies where the phase shift was close to zero for all 20 samples. Due to the negligible phase shift, these impedances can safely be considered purely resistive and characteristic to bulk osseous tissue; the capacitive impedance component of the interfaces is thus eliminated. At this frequency, the bone resistance was much higher for cortical bone samples (in the order of magnitude of kΩs) than for trabecular ones (few hundred Ωs). As expected, the phase shift was low (1° ± 2°) for both the trabecular and the cortical bone.

The morphometric parameters (Table 1) of the studied bone samples were determined based on high resolution microCT images. As expected, the cortical bone samples were similar to each other with relatively low inter-specimen standard deviations. However, the difference between the trabecular specimens was large; *e.g*., the bone volume to total volume ratio (*BV/TV*) ranged between 0.34 and 0.72 for the trabecular bone samples. The cortical bone samples were denser, with *BV/TV* values between 0.87 and 0.95. The explanation is that while cortical samples mostly contain the mineralized part of the bone, the trabecular samples used in this study represented a broad spectrum of possible bone regions.

**Table 1:** Morphometric parameters measured by microCT scans and averaged for different types of osseous tissue.

	BV/TV (—)	BS/BV (m^−1^)	TbSp m(mm)	SMI (—)	TbTh (—)	*ConnD* (mm^−3^)	*DA* (—)
Trabecular	0.53 ± 0.11	8.45 ± 1.70	0.21 ± 0.06	−2.47 ± 2.29	0.25 ± 0.05	21.6 ± 13.0	1.43 ± 0.16
Cortical	0.92 ± 0.02	1.85 ± 0.64	0.08 ± 0.02	−25.1 ± 9.4	1.20 ± 0.38	2.19 ± 2.50	2.00 ± 0.62

*BV/TV*: bone volume fraction; *BS/BV*: bone surface fraction; *TbSp*: trabecular spacing; *SMI*: structure model index; *TbTh*: trabecular thickness; *ConnD*: connectivity; *DA*: geometric degree of anisotropy.

Before establishing any relation between the morphometric and electrical properties, the correlation of the different morphometric parameters was studied (Table 2). Results showed that *BV/TV* is closely correlated to the other morphometric parameters (with Pearson’s *R*^2^ > 0.7). This observation is in alignment with the results of Maquer *et al*.^23^.

**Table 2:** Correlation matrix for the measured morphometric parameters.

*R* ^2^	BV/TV	BS/TV	TbSp	SMI	TbTh	ConnD	DA	TbN
BV/TV	1	0.96	0.91	0.74	0.73	0.35	0.21	0.67
BS/TV		1	0.78	0.76	0.79	0.49	0.16	0.81
TbSp			1	0.56	0.47	0.14	0.27	0.41
SMI				1	0.82	0.34	0.01	0.78
TbTh					1	0.44	0.02	0.87
ConnD						1	0.14	0.73
DA							1	0.08
TbN								1

Except *ConnD* and *DA*, all parameters strongly correlate to *BV/TV*.

Only two of the determined morphometric parameters could be considered independent from the bone volume fraction; these are the connectivity *ConnD* (*R*^2^ = 0.35) and the degree of anisotropy *DA* (*R*^2^ = 0.21). In addition, these two parameters were only weakly correlated to each other (*R*^2^ = 0.14).

Therefore, multi-linear regressions were conducted between the measured conductivities and the three independent morphometric parameters, *BV/TV*, *ConnD* and *DA*. Results showed that a model solely based on *BV/TV* is already able to explain the measured conductivity with a coefficient of determination of 0.83. The two other parameters only result in minor improvements in the quality of regression (improvement of *R*^2^ of less than 0.1 for each additional parameter), which indicates that these morphologic properties are not related to the measured electrical conductivity. The linear expression relating the measured conductivity *σ* to the bone volume to total volume ratio *BV/TV* is

1 $$\frac{\sigma }{{{\rm{Sm}}}^{-1}}=(0.230\pm 0.020)-(0.240\pm 0.026)BV/TV;$$

that is, conductivity decreases linearly with bone density.

**Figure 4:**
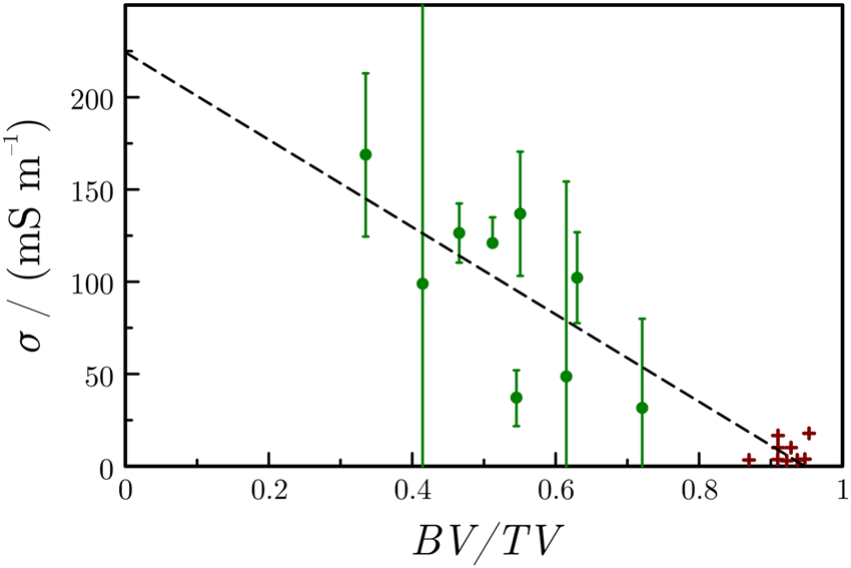
Measured bone conductivity (*σ*) at 100 kHz as a function of the *BV/TV* for the trabecular (circles) and cortical (crosses) samples. Error bars represent the standard deviation. A linear regression only based on *BV/TV* is able to closely reproduce the experimental data (*R*^2^ = 0.83).

For cortical bone (with *BV/TV* ≈ 0.92), the measured conductivity was about 9.1 mS m^−1^ (resistivity: 110 Ω m) and the conductivity of bone marrow (*BV/TV* = 0) was about 0.23 S m^−1^ (resistivity: 4.3 Ω m). By plotting the measured conductivities *vs*. the *BV/TV* of different bone samples, we gain points that fit well on the line determined by the above-mentioned two points with a root-mean-square (RMS) error of 31 mS m^−1^ for trabecular and 8 mS m^−1^ for cortical samples (Fig. 4).

## Discussion

The resistance of bone samples, measured experimentally, was compared to the bone structure determined by a microCT scanner. Results showed that the electrical properties of the bone can well be correlated to the bone volume fraction of the sample, which implies that the local electrical properties could be derived *in-vivo* from quantitative radiographic images.

Only few studies reported bone conductivity as a function of density and other parameters related to bone architecture^6,18–21^. In these studies, correlations were identified between the relative permittivity and the bone mineral density^18^, and between the dissipation factor and the *BV/TV* or *SMI*^20^. A strong linear correlation was also found between the relative permittivity and the fat content of trabecular bone. Moreover, a relationship was identified between the conductivity and the water content of trabecular bone samples^6^. In previous measurements, however, no attempt was made to distinguish between the contact surface-related impedance from the impedance corresponding to bulk bone.

In the present work, we focused on the measurement of the resistive component of the bone impedance; that is, we used impedances measured at frequencies where the phase angle was minimal, and we subsequently correlated these impedances to morphometric parameters.

**Figure 5:**
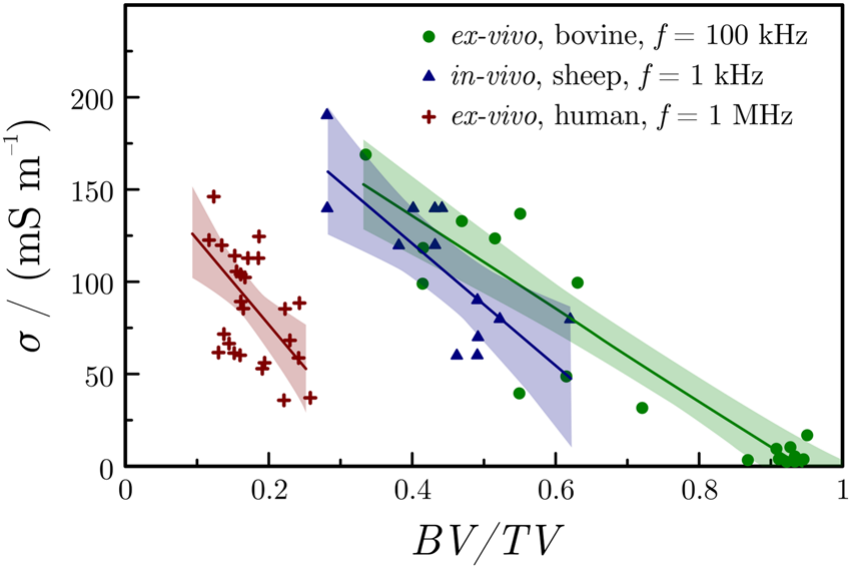
Comparison of bone conductivity (*σ*) vs. bone density (*BV/TV*) plots obtained for different bone samples. *Ex-vivo* bovine measurements were made in this study; *ex-vivo* measurements on human trabecular bone are taken from a study of Sierpowska^21^; *in-vivo* measurements on sheep mastoid bone are taken from a previous study of Wyss-Balmer *et al*.^22^.

The strength of our approach can be demonstrated by comparing our results to those of Sierpowska^21^. She reported conductivity values of samples collected on human femoral bone and, similarly to this study, found a fairly linear relationship between the conductivity and the *BV/TV* values (Fig. 5). While the intercept value (*i.e*., the conductivity of the marrow, for which *BV/TV* = 0) is similar for both experiments, the trend fitted to the data of Sierpowska^21^ predicts a conductivity of zero for *BV/TV* ≈ 0.3, which seems to be very low.

Differences between the results of Sierpowska^21^ and our results can originate either from the different nature of the bone samples (bovine *vs*. human) or from the different measuring techniques applied. In fact, in the studies of *Sierpowska et al*., impedance was determined at frequencies where the phase shift was about 10°. Here we point out that this can indicate a significant interface-related contribution in the measured impedance.

A previous study of Wyss-Balmer *et al*. quantified bone impedance *in-vivo*, using samples of sheep bone^22^. This work was performed at frequencies lower than 1 kHz but the authors proposed a model that is able to extrapolate results to higher frequencies. Interestingly, after normalization with bone density, these *in-vivo* results match relatively well with the *ex-vivo* data reported in the present study (Fig. 5). Slopes obtained from the two experiments differ slightly, which can be explained by the smaller range of densities available in the *in-vivo* study.

It is interesting to note that in their *in-vivo* experiments, Wyss-Balmer *et al*. postulated^22^ a linear relationship for resistivity *vs*. bone density, rather than for conductivity *vs*. bone density. That study focused, however, on a much narrower *BV*/*TV* range and it is thus safe to assume that a possibly non-linear (reciprocal) relationship was approximated linearly^22^. The conductivity was shown to be strongly correlated to the bone quality, which indicates that electrical measurements have the potential to inform on the local mechanical behavior of the bone.

Other studies also show a correlation between the electrical properties of the bone, like its relative permittivity, and its mechanical properties such as Young’s modulus and ultimate strength^18,19^. This confirms the potential for electrical measurements to provide insight into the mechanical performance of bone. However, unlike mechanical properties that are affected both by the bone volume fraction *BV/TV* and the fabric anisotropy *DA*^23^, electrical conductivity seems to depend solely on the bone volume fraction. Other morphometric parameters are either correlated to bone density or have no major influence on the measured conductivity.

At this point, it should be emphasized, however, that the bone samples used in this work were small and did not allow for an accurate representation of the bone anisotropy. It is likely that the importance of bone anisotropy increases when the bone region reaches an edge length of about 5 mm, which is usually the size used to estimate the bone fabric from microCT images^24,25^. Further numerical evaluations based on the conductivity derived for the marrow and the mineralized bone tissue could be used to confirm this hypothesis.

**Figure 6:**
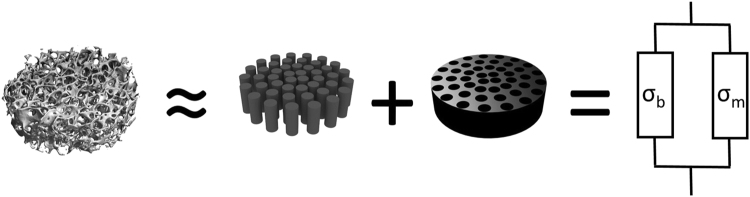
The bone samples can be described as a mixture of bone marrow embedded in mineralized structure. This situation can be modelled as an electric circuit made of two parallel idealized resistors.

To conclude, our results show that conductivity is linearly correlated to the *BV/TV*. This observation is consistent with an electrical equivalent circuit model of the bone, representing bulk osseous tissue as a parallel circuit of two resistors. One resistor (of lower resistance) accounts for bone marrow and another (of higher resistance) for bone mineral (Fig. 6).

In order to quantify this model, let us assume that our bone samples are cylinders of base area *A*. The cylinders are made of bone mineral but contain vertical channels perpendicular to the base, filled with marrow. This simplified picture allows us to assume that the portion of the base area corresponding to bone mineral (*A*_b_) and the portion corresponding to marrow (*A*_m_) can be expressed by using the bone volume fraction *BV/TV* as

2 $$\frac{{A}_{{\rm{b}}}}{A}=BV/TV\,{\rm{and}}\,\frac{{A}_{{\rm{m}}}}{A}=1-BV/TV.$$

Under these conditions it is straightforward to show that the bone conductivity is indeed a linear function of *BV/TV*:

3 $$\sigma =({\sigma }_{{\rm{b}}}-{\sigma }_{{\rm{m}}})BV/TV+{\sigma }_{{\rm{m}}},$$

where *σ*_b_ and *σ*_m_ are the conductivities of bone and marrow, respectively.

In this study, we aimed at measuring the impedance of the bone itself and therefore paid attention to remove Ringer’s solution around the samples during the measurements. However, the situation might differ during surgical intervention, since physiologic saline solution is usually used to clean the operative field or prevent tissue overheating (for example when drilling). On the other hand, we believe that the fatty bone marrow prevents the Ringer’s solution from penetrating the bone, which limits its effects to the external surfaces of the bone. This hypothesis seems to be in agreement with previous *in-vivo* measurements, where a similar bone impedance was measured, but with the bone regularly “washed” using Ringer’s solution^26^.

Cell death cannot be excluded during sample preparation, storage, and measurement. In particular, the interval of a few hours between the death of the animal and the isolation of the bone samples would have certainly resulted in extensive cell death due to hypoxia^27^. Nevertheless, care was taken to maintain cell integrity during the experiment; First, the samples were prepared using a diamond saw within a water bath, which should avoid mechanical or thermal damage to the cells. Then, all measurements were conducted quickly after the sacrifice of the animal (less than 2 days). During this time period, the samples were stored at 4 °C in Ringer’s solution, which seems appropriate to preserve cell integrity and bone electric properties^16,28^. Finally, a voltage of only 10 mV was used for the impedance measurements, which is well below the typical cell membrane potential of 60 mV to 80 mV^29^. This voltage should not be able to disrupt cellular membrane and kill cells present in the tissue. Despite the fact that cell death cannot be avoided, the similarity between our measurements and previous *in-vivo* data^26^ suggests that the preparation process preserved the electrical properties of the bone.

## Conclusion

The measurement of the impedance of biological tissue is complicated due to the presence of electrochemical effects at the interface between the metallic electrode and the sample^30^. Following the idea previously presented by Wyss-Balmer *et al*.^22^. namely that bone is a perfect resistor and that the frequency dependence is due to the double-layer interface at the point of electrode contact, we determined bone impedance at about 100 kHz, where the phase shift was approximately zero.

As a practical result, the electrical conductivity of bone was shown to be linearly correlated with the bone volume fraction *BV/TV*. Since the mechanical behavior of bone is dominated by its bone volume fraction, this result indicates that electrical measurements can be used to predict local mechanical properties of the bone. In addition, bone density can be estimated based on microCT images, which means that a map of the tissue resistivity can be directly derived from images.
